# Mouse models of pemphigus: valuable tools to investigate pathomechanisms and novel therapeutic interventions

**DOI:** 10.3389/fimmu.2023.1169947

**Published:** 2023-04-27

**Authors:** Shirin Emtenani, Michael Hertl, Enno Schmidt, Christoph Hudemann

**Affiliations:** ^1^ Lübeck Institute of Experimental Dermatology (LIED), University of Lübeck, Lübeck, Germany; ^2^ Department of Dermatology and Allergology, Philipps-Universität Marburg, Marburg, Germany; ^3^ Department of Dermatology, University of Lübeck, Lübeck, Germany

**Keywords:** pemphigus, desmoglein, autoantibody, mouse model, autoimme diseases

## Abstract

Autoimmune blistering diseases (AIBD) are paradigms of autoantibody-mediated organ-specific autoimmune disorders that involve skin and/or mucous membranes. Compared to other autoimmune diseases, the pathogenicity of autoantibodies in AIBD is relatively well described. Pemphigus is a potentially lethal autoantibody driven autoimmune disorder with a strong HLA class II association. It is mainly characterized by IgG against the desmosomal adhesion molecules desmoglein 3 (Dsg3) and Dsg1. Several murine pemphigus models were developed subsequently, each allowing the analysis of a characteristic feature, such as pathogenic IgG or Dsg3-specific T or B cells. Thus, the models can be employed to preclinically evaluate potentially novel therapies. We here thoroughly summarize past and recent efforts in developing and utilizing pemphigus mouse models for pathomechanistic investigation and therapeutic interventions.

## Introduction

Pemphigus is a severe autoimmune blistering skin disease characterized by disruption of desmosomes, thereby affecting the epidermis of the skin and surface-close mucosal epithelia (1, 2). Depending on their immunohistological manifestations, three major forms of pemphigus are distinguished. The mucocutaneous pemphigus vulgaris (PV) presents in both mucosal epithelia and the epidermis and is characterized by IgG autoantibodies (auto-abs) directed against desmoglein 1 (Dsg1) and Dsg3 antigens, which were identified over three decades ago (3). Pemphigus foliaceus (PF) is triggered by anti-Dsg1 IgG leading to intraepidermal blistering limited to the skin. Paraneoplastic pemphigus (PNP) is characterized by polymorphic mucocutaneous eruptions and originates from a pool of auto-abs mainly against plakin proteins such as envoplakin, periplakin, desmoplakins, epiplakin, desmocollins, and BP230 as well as against α2 macroglobulin-like 1 and Dsg (4, 5). In addition to Dsg3 and/or Dsg1 auto-abs, IgG auto-abs against several target proteins other than Dsgs such as desmocollin 3 were identified in pemphigus patients, raising speculations about potential synergic effects eventually triggering acantholysis (6, 7).

Autoreactive B cells are key players in the production of pathogenic antigen-specific IgG in specific organs such as the skin (8). Pathogenic auto-abs can be generated after clonal expansion of autoreactive B cells in secondary lymphoid organs, but also in the skin (9). During T-dependent and T-independent responses to Dsg3, Dsg3-specific memory B cells are generated which can remain in immunological niches and become reactivated (10). In remission following immunosuppressive therapy, these cells might potentially lead to reencounter of the clinical symptoms (11). The critical role of auto-abs in pemphigus pathogenesis is supported by the observations that i.) the level of IgG auto-abs frequently correlates with the severity of the disease (12), ii.) pemphigus-associated blister formation can be induced by passive transfer of IgG from pemphigus patients into neonatal mice (13), and iii.) transplacental transmission of pemphigus IgG antibodies from diseased mothers to neonates leading to temporal neonatal pemphigus (14).

Despite the direct pathogenic role of B cells in pemphigus, recent studies shed light on the so far largely neglected autoimmune block, the T cells. While several T-cell subsets with a strong Th2 and Th17 polarization are undoubtedly involved in the pathogenesis of pemphigus (15, 16), identifying immunodominant self-peptides and characterization of autoreactive T cells is still challenging. Indirect characterization via activation markers such as CD154 presents as a reliable method to detect antigen-reactive T cells, while other methods such as ELISPOT or incorporation of ^3^H-thymidine are unable to distinguish specific cell populations (^3^H-T, ELISPOT) or focus on a single cytokine secreted by activated T cells (ELISPOT) (17). With the advent of MHC class II–peptide technology a more comprehensive understanding of antigen-specific T cells in the immune pathogenesis of several HLA class II–linked autoimmune disorders is now achievable (18).

Considering the potential individual contributions of loss of tolerance, arising antigen-specific T and B cells as well as antigen-specific IgG as key elements of pemphigus pathogenesis, we next summarize the development of pemphigus-related mouse models focusing on those different aspects of the disease with each of the advantages and disadvantages (**Table 1**).

**Table 1:** Advantages and disadvantages of pemphigus mouse models.

Model	Mice	Advantages	Disadvantages	References
Antibody transfer-induced (“passive”)	Neonatal mice	• Faster and easier completion time • Less antibody required to induce disease • Reduction of animal usage in research	• Inability to follow up for a long period of time • Inability to produce their own auto-abs • No finalized epidermal morphogenesis • Does not allow to study of lesions in mature hair follicles and stem cell niche • No possibility of applying a systemic therapeutic approach	(13, 19, 20)
	Adult mice	• Better resembling the clinical situation • Drug screening in a therapeutic approach	• Requirement of higher amounts of antibodies to induce disease • No possibility of evaluating the role and targeted therapy of autoreactive T cells or B cells	(19, 21)
	SCID	• Investigation of autoantibody-induced pathology in human tissue	• Labor-intensive and time-consuming • Complex surgical intervention • Immunological differences between human and mouse • No further validation	(22, 23)
Lymphocyte transfer-induced	Adult mice	• Solid inducible phenotype • 10^7^ splenocytes/transfer allows a reasonable amount of recipient mice	• Artificial system due to using immune-deficient Rag2^-/-^ recipient mice	(24, 25)
Immunization-induced	Adult mice	• Active antigen-specific immune modulation • Possibility of immune interventions in both preventive and therapeutic setups	• While antigen-specific IgG total titers can be affected, no clinical phenotype is inducible so far	(26, 27)

## Animal models of pemphigus

**Figure 1:**
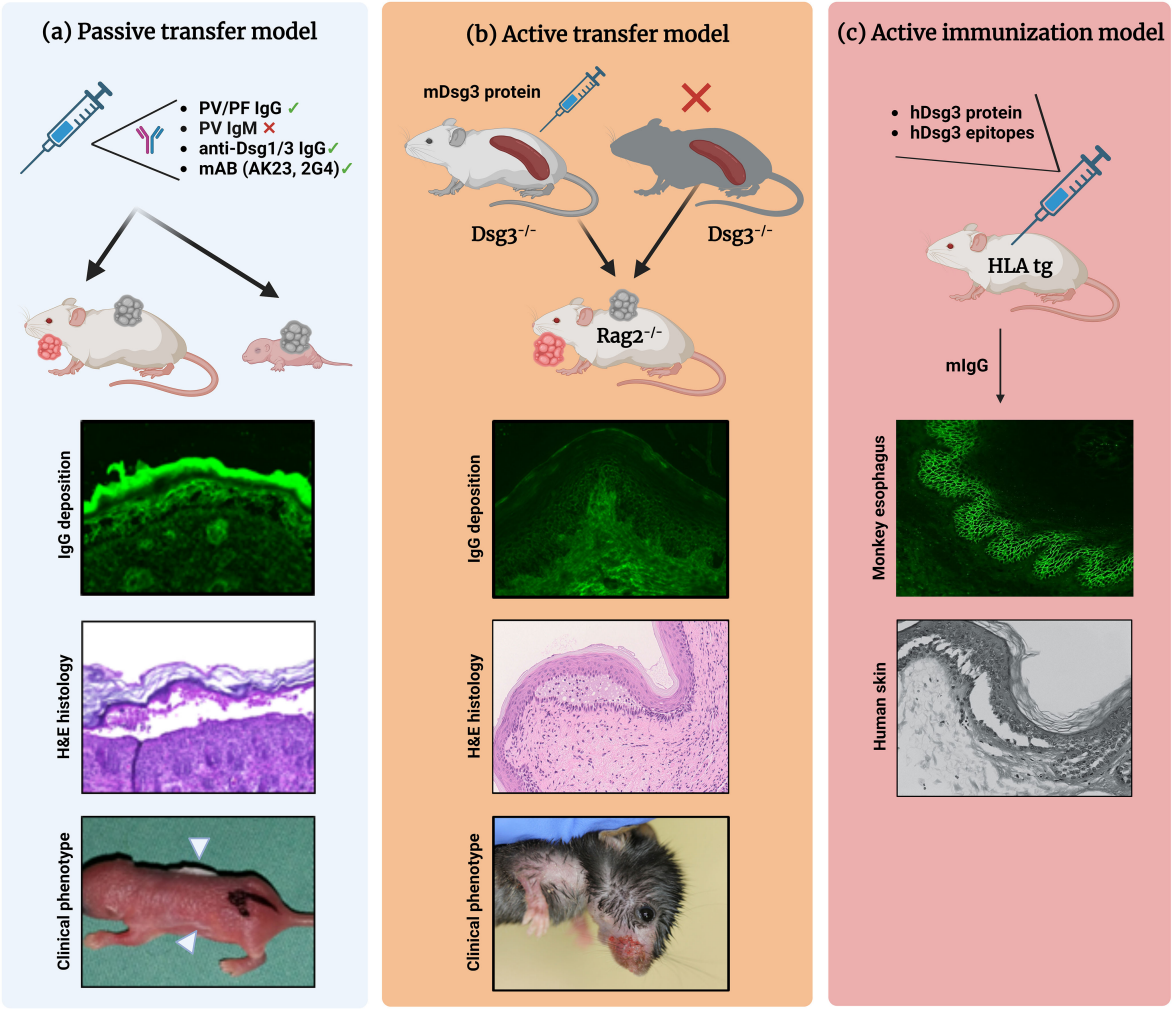
Different mouse models of pemphigus. **(A)** Antibody transfer-induced (“passive”) models. Total pemphigus vulgaris (PV) or pemphigus foliaceus (PF) IgG fractions, anti-desmoglein (Dsg) 1/3 IgG isolated from patients′ sera, or monoclonal anti-Dsg3 antibodies (e.g., AK23, 2G4) are injected into adult or neonatal mice leading to blister formation. Direct immunofluorescence (IF) microscopy, hematoxylin and eosin (H&E)-stained histology, and clinical presentation (white arrowheads indicate the blister sites) of a neonatal mouse injected with purified IgG from a patient with PV. **(B)** Lymphocyte transfer-induced models. Following adoptive transfer of naive lymphocytes from Dsg3^-/-^ mice or splenocytes from Dsg3^-/-^ mice immunized with recombinant Dsg3 protein, Rag2^-/-^ immunodeficient mice produce anti-Dsg3 IgG antibodies and display the PV phenotype, allowing us to study the loss of tolerance. **(C)** Immunization-induced models. Immunization of humanized HLA-DBR1*04:02 transgenic mice with human Dsg3 recapitulates the effector phase of T and B cells followed by the formation of pathogenic Dsg3-specific IgG formation. Binding of the murine IgG from immunized mice to the keratinocyte cell surface was confirmed by indirect IF staining on monkey esophagus. Injection into human skin with murine IgG resulted in intraepidermal acantholysis and typical pathology. Figure was created with BioRender.com.

Depending on the desired complexity of parameter induction, pemphigus-associated blister research brought forth different mouse models of pemphigus during the last three decades. *In vivo* models of pemphigus are classified as follows (**Figure 1**) (19, 28, 29):

1. Antibody transfer-induced (“passive”) models based on the injection of pemphigus auto-abs into mice to reproduce a transient disease phenotype
2. Lymphocyte transfer-induced disease models based on the transfer of autoreactive lymphocytes into mice, using transgenic models that induce a complex auto-ab-driven clinical phenotype
3. Antigen-specific immunization of mice to induce B and T cell-specific autoimmune responses including induction of pathogenic auto-abs

## Antibody transfer-induced (“passive”) mouse models

### Transfer of auto-abs into neonatal mice

The direct pathogenicity of pemphigus auto-abs has been confirmed by their passive transfer into mice. The first experimental model for pemphigus was the passive transfer model described by Anhalt and coworkers in 1982 (13). Intraperitoneal (i.p.) administration of IgG from sera of PV patients at doses of 1.5 to 16 mg/g/day into neonatal BALB/c mice resulted in cutaneous blisters and erosions with clinical, histological, ultrastructural, and immunological features of human pathology. In the course of 18-72 h following IgG administration, mice developed skin lesions in a dose- and titer-dependent manner. Continuous IgG administration led to the development of new erosions and crusts; however, skin lesions healed when the IgG injections were discontinued. This model further allowed mechanistical investigations of of auto-ab-induced acantholysis in pemphigus. In a time-course study, the ultrastructural immunological changes in the epidermis of mice injected with PV IgG were examined by electron microscopy (30). Early detachment of the epidermis was observed 1 h post IgG injection as a widening of the interdesmosomal spaces, followed by splitting of the desmosomes and complete cell detachment in the suprabasal epidermal layers within 6 h. Of note, basal cells remained unaffected in a tombstone-like pattern. Anti-Dsg3 auto-abs purified from PNP sera were also pathogenic and caused blistering in neonatal mice (31).

The principle of disease induction by passive transfer of pathogenic PV IgG into neonatal mice has also been successfully applied to other pemphigus variants. Roscoe et al., in 1985 published the first *in vivo* study on the pathogenic role of anti-Dsg1 antibodies (32). When injected into neonatal mice, PF IgG (at 10 mg/g/day for a total of four injections) induced superficial skin blisters reproducing the clinical and immunopathological findings of human disease. Similar to the PV model (13), there was a close correlation between disease severity and the amount of injected IgG. In contrast to PV IgG, PF IgG did not cause suprabasal acantholysis in the epidermis but rather a superficial loss of epidermal adhesion, suggesting that PV and PF are mediated by different auto-abs specificities. Further studies showed that the pathogenic auto-abs in pemphigus are predominantly of the IgG4 subclass (33, 34). In contrast, some PF patients only had IgG1 auto-abs, which caused acantholysis in mice (35).

### Autoantibodies alone cause skin pathology in pemphigus

Unlike the pemphigoids, IgG-induced acantholysis in pemphigus is independent of complement activation or Fc-effector function. In an experimental passive transfer model, i.p. injection of intact pathogenic IgG and F(ab′)2 from PV sera led to the same extensive blistering, while the latter failed to induce C3 deposition. In addition, blistering was observed in C5-deficient or cobra venom factor (a structural analog of complement component C3) pretreated neonatal mice following administration of PV IgG (36). Hence, it was speculated that crosslinking of PV antigen on the surface of keratinocytes by bivalent PV auto-abs may be a necessary step for acantholysis.

Another study showed that subcutaneous (s.c.) injection of F(ab′)2 and Fab fragments caused blistering in mice (37). Pemphigus-like lesions have also been induced in neonatal mice by passive transfer of F(ab′)2 and Fab′ fragments purified from PF IgG (33).

These early studies relied predominantly on polyclonal IgG or its cleavage products. Although patient’s IgG is certainly an excellent tool for investigation of pemphigus pathomechanisms, its limited availability and heterogeneity over the course of the disease and between individuals may complicate systematic studies (38, 39). Monoclonal antibodies (mAbs), developed by immunization of mice with the recombinant mouse Dsg3 ectodomain, such as AK23, or isolated by phage display from active pemphigus patients, are now being extensively used to model PV pathogenesis (40–44). Payne et al. cloned anti-Dsg3 mAbs as single-chain variable fragments (scFvs) from a mucocutaneous PV patient using antibody phage display, which induced typical PV lesions in neonatal mice (40). Using the same technique, Ishii et al., isolated anti-Dsg1 scFv mAbs from a PF patient that were pathogenic in mouse and human skin (44). While most pathogenic Dsg3-related mAbs bind to the membrane distal EC-1/2 regions of the Dsg3 ectodomain, a novel Dsg3-EC5 binding mAb 2G4 allows the characterization of membrane proximal pathogenic binding (45). Additionally, experimental pemphigus can also be induced by Dsg3-hybridoma cell lines. Passive transfer of AK23-IgG hybridoma derived from splenocytes of the PV model caused blistering in neonatal mice (41). The practical advantage of hybridoma technology is that once stable somatic cell lines are established, they can be used to produce sensitive and specific mAbs in unlimited quantities. Furthermore, this technique preserves the native pairing of antibody variable and constant regions (46). Despite these advantages, hybridoma cells are limited by long generation times and the difficulty of controlling the epitopes against which antibodies are produced. Immunoadsorption of pathologic auto-abs from PV sera using the entire EC domains of Dsg1 and Dsg3 abolished the blister-inducing ability of IgG fractions in mice, suggesting that anti-Dsg1/3 IgG alone is pathogenic; other factors such as additional non-desmoglein PV auto-abs alone or in combination are not required (47, 48). In line with these observations, adjuvant immunoadsorption has been successfully applied in patients with severe PV and PF to efficiently reduce circulating pathogenic auto-abs (49–51).

The possible involvement of IgM in the pathogenesis of PV has also been investigated. In experimental PV, in contrast to AK23 IgG, administration of its AK23-IgM hybridoma cells isotype did not induce clinically overt blisters (52). As monovalent (Fab), bivalent (F(ab)’)2), or scFv fragments of anti-Dsg auto-abs induce keratinocyte dissociation *in vitro* and *in vivo*, the Fc portion of pathogenic pemphigus auto-abs is probably of minor significance (53).

Taken together, these results demonstrate that Fc-mediated mechanisms are not required for blister formation in PV and PF. Accordingly, research in pemphigus has focused on characterizing anti-Dsg B cells and antibodies as well as signaling pathways that modulate the pathogenic effects of auto-abs.

### Desmoglein compensation model explains the localization of lesions

Dsg1 or Dsg3 can, at least in part, compensate for the adhesive functions of each other (1, 54). The desmoglein compensation model has been supported by several experimental studies. Passive transfer of PF IgG to neonatal mice caused loss of adhesion in the superficial epidermis (55). Consistent with this finding, exfoliative toxins produced by *Staphylococcus aureus* specifically cleave Dsg1, resulting in blistering just below the stratum corneum of neonatal mice (56, 57). On the other hand, forced superficial expression of Dsg3 in transgenic mice protected them from PF antibody-induced blistering. Another proof comes from the passive transfer of pemphigus IgG to normal and neonatal Dsg3*^null^* mice. Basically, Dsg3*^null^* neonatal mice do not present skin blisters because Dsg1 is present throughout the epidermis to compensate for the genetic loss of Dsg3. However, challenging these mice with anti-Dsg1 IgG led to severe PV-like blistering similar to cutaneous PV patients (54, 58). Additionally, the telogen hair sheds early in Dsg3^−/−^ mice leading to an alopecia areata-like phenotype, whereas transgenic Dsg1 over-expression in the telogen club leads to a decreased or delayed balding phenotype (59).

## Emerging novel therapeutic targets for pemphigus

### Targeting signaling pathways

Beyond steric hindrance, signaling pathways induced by binding of pemphigus IgG have been proposed to indirectly trigger acantholysis (60–62). Pharmacological modulation of signaling molecules blocked blister formation in the passive transfer mouse models for pemphigus. Consequently, some of the therapies derived from treating these experimental models might be translatable to human disease. For instance, 38 mitogen-activated protein kinase (p38MAPK) was shown to be activated by polyclonal PV (63, 64) and PF IgG (65) *in vivo*. Furthermore, pretreatment with p38MAPK inhibitors (i.e., SB202190 and SB203580) blocked PV and PF IgG-induced blistering in mice, suggesting a pivotal role for p38MAPK signaling in acantholysis (64–66). On the other hand, blisters induced by mAbs isolated from PV patients were not affected by p38 or MAPK-activated protein kinase 2 (MK2) inhibition, pointing to the significance of steric hindrance mechanisms (42, 67). Application of a Dsg3-specific tandem peptide stabilized adhesion inhibited PV IgG-mediated activation of p38MAPK and skin blistering (68). Unfortunately, clinical trials with a p38MAPK inhibitor (NCT00606749) in PV patients were terminated due to its dose-limiting hepatotoxicity (69). Downstream of p38MAPK, epidermal growth factor receptor (EGFR) signaling was shown to be activated in PV IgG-treated keratinocytes. In line, treatment with AG1478, an inhibitor of EGFR signaling, blocked blister formation in mice (70). Moreover, other clinically approved EGFR (Erlotinib) and dual EGFR/ErbB2 (Lapatinib) inhibitors prevented blistering in a neonatal PV mouse model (71). ADAM10 together with Src regulates signaling downstream of Dsg3 which may also include EGFR signaling. It was shown that PV IgG increased ADAM10 activation in a Src-dependent manner. Thus, inhibition of ADAM10 prevented histological acantholysis and skin blistering in mice injected with PV IgG (72). In addition, in neonatal mice injected with PV IgG, enhanced phosphorylation of mammalian target of rapamycin (m-TOR) and Src as well as increased blister formation and apoptosis were observed, all of which were eliminated or reduced by focal adhesion kinase (FAK) inhibition (73). In another experimental setting, modulation of mTOR with sirolimus (or rapamycin) and Src with PP1 abolished acantholysis (74). Additionally, Src blockade was protective against AK23-induced skin blistering (75). In a neonatal PV model, pharmacological inhibition of cyclin-dependent kinase 2 (Cdk2) by roscovitine prevented blister formation (76). Pretreatment of mice with inhibitors of tyrosine-kinase (TK) completely prevented the clinical and histological findings of PV (77). PV IgG was demonstrated to induce protein kinase C (PKC) activation as well. In line with these findings, PKC inhibition reduced PV IgG-induced skin blistering *in vivo* (78, 79). Finally, elevated levels of cyclic adenosine monophosphate (cAMP) have been shown to interfere with signaling pathways and prevent blister formation *in vivo* (80). A recent study showed that the phosphodiesterase 4 (PDE4) inhibitor apremilast inhibited blister formation in a passive transfer PV model via protective cAMP signaling in keratinocytes. Hence, apremilast offers a promising approach to target loss of desmosomal adhesion in pemphigus patients (81). PV IgG not only induces direct signaling effects but also changes gene expression patterns, which may contribute to pemphigus pathogenesis. In contrast, corticosteroids antagonistically upregulate *DSG3* transcription through inhibition of the transcription factor Stat3 (82, 83). Additionally, it has been demonstrated that methylprednisolone reduces the extent of PV IgG-induced acantholysis in the skin of neonatal mice.

### Targeting Fas ligand

Several lines of evidence indicate that certain mediators involved in apoptosis contribute to the pathological mechanisms of pemphigus, while apoptosis itself is not apparent in pemphigus lesions nor required for acantholysis *in vitro* (84–86). In particular, cleaved caspase-8 and -3 were detected in pemphigus lesions, and upregulation of Fas ligand (FasL) was observed in keratinocytes co-cultured with PV IgG (87–89). Accordingly, a study by Lotti et al., showed that an anti-FasL antibody blocked blister formation in a neonatal mouse model of PV. Following PV IgG injection, sFasL^-/-^ mice (lacking secreted soluble form of FasL) revealed reduced acantholytic area, whereas mFasL^-/-^ mice (lacking membrane-bound form of FasL) developed blisters. These observations indicate that sFasL, which is upregulated and released from keratinocytes, plays a critical role in pemphigus pathogenesis (90). In a neonatal PF model, the administration of caspase inhibitors blocked intraepidermal blistering (91). In line, a novel anti-sFasL human mAb (PC111) has been tested for pemphigus therapy due to low its potential for immunogenicity, favorable chemical and physical stability, and high binding affinity of the compound (92).

### Targeting neonatal Fc Receptor

Several studies have shown a pivotal role for FcRn in pemphigus. Li et al., demonstrated that FcRn-deficient mice were resistant to experimental PV and PF, in which circulating auto-ab levels were significantly reduced as compared to wild type mice. Besides, the therapeutic efficacy of high-dose intravenous immunoglobulins (IVIg) might be attributed to FcRn saturation and increased catabolism of pathogenic IgG (93). Of note, IVIg protected target cells from apoptosis by interfering with signaling pathways and increasing the sensitivity to corticosteroids (89). In an auto-ab-induced model of PV, IVIg selectively inhibited anti-Dsg3 antibodies, decreased the number of circulating auto-abs, and reduced blister formation (94). Additionally, disease-specific IVIg was shown to be more effective in neutralizing pathogenic antibodies and preventing blister formation *in vivo* than commercial IVIg (95). Clinical trials are currently evaluating the safety and efficacy of several FcRn‐targeting compounds (e.g., efgartigimod (ARGX-113) and SYNT001 (ALXN1830)) in pemphigus (96–98). Showing unexpected effects for efgartigimod *in vitro*, a recent study additionally suggested that efgartigimod-induced blockade of FcRn may have functions on B cell homeostasis beyond IgG recycling. Improvement of keratinocyte adhesion by FcRn blockade may provide a novel treatment option for pemphigus (39). However, the antibody-induced model is not suitable to study the role and specific targeting of autoreactive T cells or B cells.

### Transfer of autoantibodies into adult mice

Due to incomplete epidermal development in neonates, the principle of autoantibody transfer has been further adapted to adult mice. Experimental PV was induced in adult C57Bl/6J or Rag2^-/-^ mice by passive transfer of a murine pathogenic Dsg3 mAb, AK23 (21). This model has proved useful in exploring non-apoptotic aspects of pemphigus related signaling. For instance, EGFR was shown to be activated in response to AK23, followed by an increase in c-myc levels. Adult passive transfer model of pemphigus was also applied in fully humanized Dsg3 (hDsg3) mice, showing that the administration of anti-Dsg3 serum IgG from patients with mucosal PV (mPV) caused IgG deposits on the surface of epidermal keratinocytes and suprabasilar acantholysis in mucosal tissues of hDsg3 mice, but not in WT mice. This finding confirms the *in vivo* pathogenicity of mPV anti-hDsg3 IgG (99).

### Transfer of autoantibodies into human skin grafted onto mice

An alternative approach for studying the pathogenesis of human auto-abs in mice is to passively transfer pemphigus serum into athymic nude mice grafted with human oral mucosa. This model combines a mouse model with human skin allowing *in vivo* studies with pathogenic anti-human Dsg3 auto-abs (22, 100). A low degree of epithelial cell detachment was observed in this mucosal graft model, whereas human IgG was detected in the skin of all mice, and nearly two-thirds of the transplants showed basal epithelial edema (23). When full-thickness human skin was grafted onto the backs of severe combined immunodeficient (SCID) mice followed by injection of PF and PV IgG, subcorneal and suprabasal loss of adhesion as well as intercellular IgG deposits in the upper and lower layers of the epidermis, respectively, was seen (22). Because the xenograft model in pemphigus is complex and adds little to a classical passive model, few experiences have been gained with these models.

## Lymphocyte transfer-induced disease models based on the transfer of autoreactive lymphocytes into mice

Considering the half-life of serum IgG, passive transfer models can only be used and analyzed for a limited amount of time. The main obstacle to an active immunization model is immunological self-tolerance. The usage of pemphigus mouse models was transformed by the development of Dsg3^-/-^ knockout mice. These mice show a loss of keratinocyte cell adhesion mainly in oral mucous membranes, a phenotype that resembles that of patients with PV (24). These erosions, also occasionally found in the skin with suprabasal acantholysis demonstrated the importance of Dsg3 *in vivo*, however, no immunological inherent components were involved. Immunization with murine Dsg3 induced a more severe phenotype in terms of affected skin area and eosinophilic spongiosis (25). To further pinpoint Dsg3-specific immune modulation, immunization of Dsg3^-/-^ mice with recombinant murine Dsg3 was followed by the intravenous transfer of the splenocyte pool into immunodeficient Rag2^-/-^ Dsg3^+/+^ recipient mice (25). Newly formed antigen-specific T and B cells then induced a Dsg3-specific autoimmune response presenting with suprabasal acantholysis, rows of tombstone basal keratinocytes, and half-desmosomes (101). Interestingly, antigen-specific T cell infiltration into Dsg3-expressing tissues led to interface dermatitis, a distinct form of T cell-mediated autoimmunity causing acantholysis and can be found in autoimmune skin diseases such as PNP (102). Establishing a retroviral transduction system, Takahashi et al., generated a C57BL/6J-Tg (Dsg3TCR140) mouse line which enabled them to show that while tolerized Dsg3H1 T cells could induce interface dermatitis, but not PV, non-tolerized Dsg3H1 T cells induced both anti-Dsg3 IgG production and interface dermatitis. These results demonstrated that induced anti-Dsg3 auto-abs potentially interfere with cell-cell adhesion of keratinocytes in the PV model. Based on those studies, a series of human, murine, and bispecific T cell clones were generated. Further characterization indicated that although an individual anti-Dsg3 IgG is not sufficient to cause acantholysis in adult mice, several clones together can induce a pemphigus phenotype (43). An array of various immunosuppressive agents frequently used in PV patient treatment was evaluated by means of ELISA, weight, and PV severity score (103). While cyclophosphamide displayed the strongest immunosuppressive properties, other agents such as azathioprine were shown to be less effective or not suppressive at all such as methylprednisolone and dexamethasone. Those findings are partially in contrast to the experience in humans (104), showing that observations in preclinical mouse models cannot be directly transferred to the human situation. Unfortunately, no follow-up studies applying novel treatment modalities in this model have been published so far.

Another modification of the pemphigus mouse model involved the transfer of naїve splenocytes from Dsg3^-/-^ mice into Rag2^-/-^ Dsg3^+/+^ recipients (105). Here, Dsg3-specific naїve lymphocytes in Dsg3^-/-^ mice can be primed and activated by endogenous Dsg3 in recipient mice to ultimately induce pathogenic anti-Dsg3 IgG without active immunization. While the time and overall levels of IgG and phenotype induction were delayed by two weeks, once the phenotype developed, no apparent differences in disease severity between Rag2^-/-^ recipients of naïve or immunized splenocytes were found (105). Immunosuppressive drugs such as cyclophosphamide successfully inhibited disease development in a preventive setting, and mice were free of symptoms 35 days after discontinuing the treatment (106). To further pinpoint crucial CD4^+^ subpopulations for anti-Dsg3 IgG formation, Kim et al., developed a modified murine transfer protocol based on Rag1^-/-^ recipient mice and provided compelling data that Dsg3-specific ICOS^+^ T follicular helper (Tfh) cells play a pivotal role in pathogenic humoral immunity in PV (107). Since alterations in the Tfh compartment have previously been found in PV (108), targeting ICOS represents a valid therapeutic option for the treatment of PV.

## Antigen-specific immunization of mice to induce B and T cell-specific autoimmune responses including induction of pathogenic antibodies

PV is an oligoparametric disease, arising from a combination of different environmental factors in predisposed individuals carrying individual susceptibility genes. While genome-wide association studies are lacking to complete the picture of protective, susceptible, or neutral alleles (109), two highly associated class II alleles have been found in PV patients (DQB1*0503 and DRB1*0402) (110). PV patients carrying these alleles showed Dsg3-specific auto-aggressive Th2 cells, while healthy carriers preferentially displayed an autoreactive Th1 response (111). This prompted us to develop an active mouse model using a C57B/6J transgenic mouse line with the respective alleles HLA-DQB1*04:02 in a linkage disequilibrium with HLA-DQB1*03:02 that additionally expresses the human CD4 co-receptor while lacking a functional murine major histocompatibility class II molecule (I-Aβ−/−) (26). Antigen presentation after Dsg3 immunization is therefore restricted to human alleles which are presented in association with these human HLA class II alleles to CD4+ T cells. Intraperitoneal immunization with human Dsg3 protein or with a set of immunodominant Dsg3-peptides (50 µg/mouse day 0 and day 14), which share a positively charged anchor motif for HLA-DRB1*04:02, led to the formation of Dsg3-reactive CD4^+^ T cells followed by a profound and lasting induction of anti-Dsg3 IgG (26). This model was used to show that T and B cell interaction is crucial for pemphigus pathology. Blockade of this interaction by anti-CD40L mab completely prevented anti-Dsg3 IgG formation. Furthermore, the induction of T regulatory cells by the superagonistic anti-CD28 antibody D665 also significantly reduced anti-Dsg3 IgG induction (112).

Dsg3-specific immunization of the HLA tg mice allows cellular characterization and therapeutic intervention studies in a preclinical setting (i.e., during the formation of antigen-specific T and B cells and subsequent auto-Ab formation) or a therapeutic setting (i.e., after the onset of immunization with an ongoing auto-Ab formation as in human PV). Using this model, CD4+ T cell-specific tolerance could be induced and analyzed based on the application of a set of immunodominant Dsg3 CD4+ T cell epitopes linked to nanoparticles (113). For identification and characterization of low-frequent Dsg3-specific CD4+ T cells in lymphatic tissues, a novel detection based on DRB1*04:02 HLA class II specific dextramers loaded with the aforementioned immunodominant Dsg3-T cell epitopes was developed. CD4+ T cells were primarily detected in lymphoid tissue after initial immunization (4% Dsg3+ CD4+ CD19+ cells) which steadily decreased thereafter. Additionally, applying IVIg as an established treatment for pemphigus was shown to modulate antigen-specific T and B cell formation (114). A significant reduction of Dsg3-specific serum IgG correlated with an upregulation of regulatory T cells.

Recent endeavors to break tolerance against Dsg3 in various strains of Dsg3-expressing mice employing different immunization protocols with recombinant human and murine Dsg3/Dsg1 forms did not elicit a clinical phenotype while non-pathogenic anti-Dsg3/Dsg1 IgG was induced (115). However, unpublished work currently focuses on the establishment of a Dsg3-transgenic mouse model displaying the formation of HLA-dependent antigen-specific T and B cells as well as a solid antigen-specific IgG as a basis of a lasting clinical phenotype. This model will be suitable for in-depth analysis of autoreactive B and T cells and IgG formation as well as a preclinical model for testing novel specific immune interventions in pemphigus.

## Conclusions

Even though our understanding of T- and B-cell-related induction of antigen-specific IgG in AIBD PV and PF has dramatically improved over the last decades, we still lack a proper mouse model that reflects the “grande picture” of patient-relevant characteristics. The interplay between blister formation based on steric hindrance by auto-abs against Dsg3 and prominent induced signaling pathways such as p38MAPK or ERK is still under debate. The identification of novel PV-related antigens allows the further distinction of novel clinical subtypes with specific clinical outcomes. Different preclinical models reflect distinct hallmarks and pathways of PV immune pathogenesis, i.e., induction of autoreactive T and B cells and auto-ab-induced acantholysis. They are constantly improved and reflect, so far, only a few PV-related parameters. Novel therapeutic interventions in PV address these parameters and be further characterized *in vivo*. Nonetheless, a mouse model reproducing PV pathology based on HLA-dependent T- and B-cell mediated antibody formation leading to mucosal acantholysis would be a desired gold standard.

## Author contributions

All authors contributed to the writing and review of this manuscript. All authors approved the final version of this manuscript. All authors contributed to the article and approved the submitted version.
